# Evaluation of MC3T3-E1 Cell Osteogenesis in Different Cell Culture Media

**DOI:** 10.3390/ijms22147752

**Published:** 2021-07-20

**Authors:** Makoto Izumiya, Miyu Haniu, Katsuya Ueda, Haruka Ishida, Chuang Ma, Hirokazu Ideta, Atsushi Sobajima, Koki Ueshiba, Takeshi Uemura, Naoto Saito, Hisao Haniu

**Affiliations:** 1 Institute for Biomedical Sciences, Interdisciplinary Cluster for Cutting Edge Research, Shinshu University, 3-1-1 Asahi, Matsumoto, Nagano 390-8621, Japan; 21hb401k@shinshu-u.ac.jp (M.I.); oceangrace326h@yahoo.co.jp (M.H.); 19hb402j@shinshu-u.ac.jp (K.U.); 18hb401g@shinshu-u.ac.jp (H.I.); 20hb403b@shinshu-u.ac.jp (C.M.); 21bs201b@shinshu-u.ac.jp (K.U.); tuemura@shinshu-u.ac.jp (T.U.); saitoko@shinshu-u.ac.jp (N.S.); 2 Biomedical Engineering Division, Graduate School of Medicine, Science and Technology, Shinshu University, 3-1-1 Asahi, Matsumoto, Nagano 390-8621, Japan; ideta@shinshu-u.ac.jp; 3 Department of Orthopaedic Surgery, School of Medicine, Shinshu University, 3-1-1 Asahi, Matsumoto, Nagano 390-8621, Japan; soba@shinshu-u.ac.jp; 4 Department of Orthopedics (Lower Limbs), Social Medical Care Corporation Hosei-kai Marunouchi Hospital, 1-7-45 Nagisa, Matsumoto, Nagano 390-8601, Japan; 5 Biomedical Engineering Division, Graduate School of Science and Technology, Shinshu University, 3-1-1 Asahi, Matsumoto, Nagano 390-8621, Japan; 6 Research Center for Supports to Advanced Science, Division of Gene Research, Shinshu University, 3-1-1 Asahi, Matsumoto, Nagano 390-8621, Japan

**Keywords:** biomaterials, vitamin C, osteoblast, calcification, cell culture, collagen, alkaline phosphatase

## Abstract

Many biomaterials have been evaluated using cultured cells. In particular, osteoblast-like cells are often used to evaluate the osteocompatibility, hard-tissue-regeneration, osteoconductive, and osteoinductive characteristics of biomaterials. However, the evaluation of biomaterial osteogenesis-inducing capacity using osteoblast-like cells is not standardized; instead, it is performed under laboratory-specific culture conditions with different culture media. However, the effect of different media conditions on bone formation has not been investigated. Here, we aimed to evaluate the osteogenesis of MC3T3-E1 cells, one of the most commonly used osteoblast-like cell lines for osteogenesis evaluation, and assayed cell proliferation, alkaline phosphatase activity, expression of osteoblast markers, and calcification under varying culture media conditions. Furthermore, the various media conditions were tested in uncoated plates and plates coated with collagen type I and poly-L-lysine, highly biocompatible molecules commonly used as pseudobiomaterials. We found that the type of base medium, the presence or absence of vitamin C, and the freshness of the medium may affect biomaterial regeneration. We posit that an in vitro model that recapitulates in vivo bone formation should be established before evaluating biomaterials.

## 1. Introduction

Biomaterial development has traditionally focused on improving surface modifications and shape-processing technology for hard-tissue implant materials in orthopedics and dental surgery [1,2,3]. Although many materials offer excellent physical, chemical, and engineering properties with respect to biological safety, only few have been commercialized for clinical applications. Cost is a possible underlying reason; however, in some instances, in vitro and in vivo evaluations are inconsistent [4]. According to such findings, existing in vitro functional evaluation methods do not reflect the bone remodeling and healing processes in vivo.

In vitro biological evaluation of the effects of implant materials on bone tissue has been mainly based on osteogenic potential using cultured osteoblasts, from primary cells to cell lines, including induced pluripotent stem cells (iPSCs). While primary cells reportedly reflect the biological state more faithfully, they are difficult to validate. In contrast, cell lines are more homogeneous. Further, experiments using iPSCs are costly, and homogeneity with respect to biological responses is limited in cells other than iPSCs [5,6]. In both cases, the culture medium is a factor affecting the evaluation. Although the importance of culture medium has been previously demonstrated for certain osteoblast experiments [7,8], little attention has been paid to culture media conditions when evaluating the hard-tissue regeneration ability of biomaterials in vitro.

Ascorbic acid is especially important when culturing osteoblasts. Vitamin C (VC) is the L-enantiomer of ascorbic acid and an essential nutrient for living organisms. However, while animals such as rats and mice can biosynthesize VC, humans and some monkeys cannot; thus, VC must be obtained from external sources. VC deficiency causes scurvy due to lack of normal collagen synthesis, and its importance in bone tissue has also been demonstrated [9,10]. Several protocols have been developed wherein culture media containing VC are used for the primary culture of osteoblasts [11,12]. However, several primary culture methods that use media without VC exist [13,14], and there are inconsistencies in the media used for primary cultures. This contradiction also exists in osteoblast cell lines. The MC3T3-E1 mouse calvaria-derived preosteoblast cell line established in Japan [15] has contributed remarkably to the investigation of the role of osteoblasts in bone formation [16,17]. The Japanese supplier Riken BRC recommends culturing these cells with α minimum essential medium (αMEM) containing VC. Conversely, based on a report by Wang et al., the American Type Culture Collection (ATCC) recommends using αMEM without VC [18]. Surprisingly, VC has been added as a differentiation-inducing factor along with glycerophosphate—a source of phosphate—to primary cultured and iPSC-derived osteoblasts cultured in media with or without VC to induce calcification and study osteoblast osteogenesis [19,20]. These inconsistencies indicate that the results of functional assessment of osteoblasts may differ depending on the conditions in the medium itself.

Few reports have examined the effects of differences in media on the efficacy of biomaterials with respect to regeneration. In this study, we first evaluated the effects of varying culture media on proliferation and osteogenesis in the well-characterized MC3T3-E1 cell line. We then evaluated culture plates with different surface treatments in different media to clarify the effects of media conditions on biomaterial evaluation. This study is an essential first step in designing an in vitro model that accurately reflects the in vivo modeling process in hard-tissue implant development.

## 2. Results

### 2.1. Cell Proliferation

**Figure 1:**
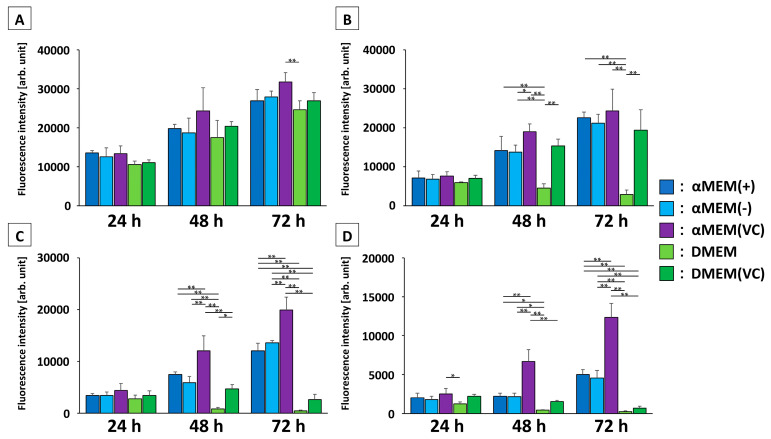
Proliferation of MC3T3-E1 cells in different media with initial cell density: (**A**) 60 × 10^3^ cells/mL; (**B**) 30 × 10^3^ cells/mL; (**C**) 15 × 10^3^ cells/mL; (**D**) 7.5 × 10^3^ cells/mL. Proliferation is represented as mean fluorescence intensity ± SD (*n* = 4, * *p* < 0.05; ** *p* < 0.01). Multiple group comparisons between different media were made daily.

We first tested the capacity of cells to proliferate using the viability dye AlamarBlue by seeding them at varying densities and tracking their growth in the different culture media: αMEM containing VC (αMEM (+)) or without VC (αMEM (-)), αMEM (-) supplemented with VC (αMEM (VC)), DMEM, or DMEM supplemented with VC (DMEM (VC)). At the highest initial cell density, MC3T3-E1 cells proliferated under all media conditions; however, there was a significant difference after 72 h between αMEM (VC), which showed the highest cell proliferation, and DMEM, which showed the lowest (*p* < 0.01, Figure 1A). Moreover, MC3T3-E1 cells cultured in DMEM could not proliferate at a concentration below 30 × 10^3^ cells/mL (Figure 1B). At densities below 15 × 10^3^ cells/mL, MC3T3-E1 cells did not proliferate in DMEM (VC) (Figure 1C,D). Conversely, MC3T3-E1 cells proliferated regardless of the initial cell density in an αMEM-based medium. However, at initial densities below 15 × 10^3^ cells/mL, MC3T3-E1 cells showed significantly higher proliferation in αMEM (VC) after 48 h than they did in αMEM (+) or αMEM (-). There was no significant difference in the proliferation of MC3T3-E1 cells cultured in αMEM (+) and αMEM (-) at any initial cell density.

### 2.2. Alkaline Phosphatase (ALP) Activity

**Figure 2:**
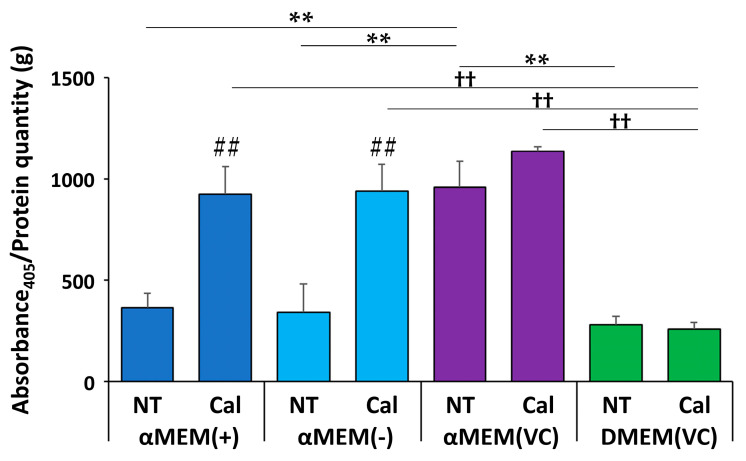
Quantitative results of alkaline phosphatase (ALP) activity in MC3T3-E1 cells 7 d after calcification induction. ALP activity is expressed as Abs_405_/g protein (*n* = 3, ** *p* < 0.01, ^††^ *p* < 0.01, ## *p* < 0.01): ** multiple group comparisons among nontreated (NT) media; ^††^ multiple group comparisons among calcification (Cal) media; ## two-group comparison between NT and Cal media.

Measured ALP activity 7 d after induction of calcification differed depending on media conditions (Figure 2). MC3T3-E1 cells cultured in αMEM (VC) had more than twice the amount of ALP activity compared to cells cultured in other media without calcification induction, and there was no significant difference between αMEM (+), αMEM (-), and DMEM (VC). When calcification was induced in MC3T3-E1 cells, the groups showed two different media-dependent responses. αMEM (VC) and DMEM (VC) did not show any increase in ALP activity upon calcification induction; however, αMEM (+) and αMEM (-) showed a similar increase in ALP activity, which was almost the same as nontreated (NT) αMEM (VC).

### 2.3. Calcification

**Figure 3:**
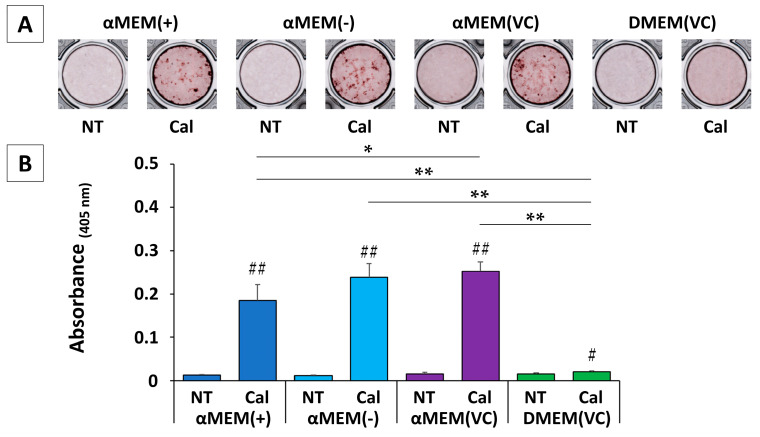
Calcification in MC3T3-E1 cells after 21 d: (**A**) representative images of Alizarin Red S (ARS) staining in various media in nontreated (NT) vs. calcification (Cal) conditions. (**B**) Solutions of mineral nodules eluted by agitation with 5% formic acid for 10 min were measured and quantified by absorbance at 405 nm (*n* = 4, * *p* < 0.05, ** *p* < 0.01, # *p* < 0.05, ## *p* < 0.01): * multiple group comparison among calcification (Cal) media; # two-group comparison between NT and Cal media.

We stained MC3T3-E1 cells with Alizarin Red S (ARS) three weeks after the induction of calcification. Typical images for each group are shown in Figure 3A. ARS was subsequently redissolved in formic acid and quantified (Figure 3B). All cultured MC3T3-E1 cells showed slight staining in any non-treated (NT) medium, but cells cultured in an αMEM-based medium under calcification induction conditions showed marked calcification. In addition, cells cultured in αMEM (VC) showed significantly higher calcification than those cultured in αMEM (+) (*p* < 0.05). Conversely, in DMEM (VC), while calcification induction resulted in significantly higher calcification (*p* < 0.05) than in NT media, this increase was extremely small compared to the response to calcification induction in an αMEM-based medium. Cells cultured in DMEM (VC) showed almost no calcification at three weeks, but calcification was notable at five weeks (Figure S1).

### 2.4. Gene Expression

**Figure 4:**
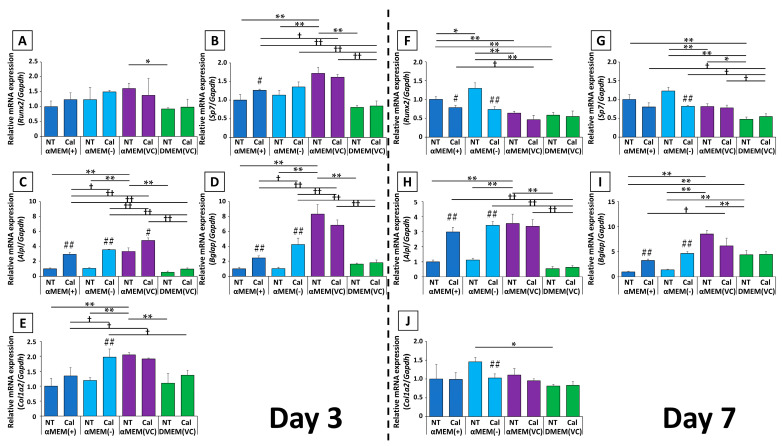
Comparative qPCR for (**A**,**F**) *Runx2*, (**B**,**G**) *Sp7*, (**C,H**) *Alpl*, (**D**,**I**) *Bglap*, and (**E**,**J**) *Col1a2* on days 3 and 7 after calcification induction. Target-gene expression was normalized to *Gapdh* expression and was calculated by comparing the expression in MC3T3-E1 cells cultured in nontreated αMEM (+) (*n* = 3, * *p* < 0.05, ** *p* < 0.01, ^†^ *p* < 0.05, ^††^ *p* < 0.01, # *p* < 0.05, ## *p* < 0.01): * multiple group comparison among nontreated (NT) media; ^†^ multiple group comparison among calcification (Cal) media; # two group comparison between NT and Cal media.

Transcript-level expression of several osteoblast markers after calcification induction was assessed at days 3 and 7, relative to expression in MC3T3-E1 cells cultured in αMEM (+) without induction (Figure 4).

The expression of the early osteoblast transcription factors *Runx2* and *Sp7* showed similar changes. In αMEM (+) and αMEM (-), calcification induction resulted in increased expression at day 3, followed by decreased expression by day 7. Meanwhile, in αMEM (VC) and DMEM (VC), there was little change in the expression of these genes at days 3 or 7. In addition, the expression ratio among untreated media on day 3 was higher in cells cultured in αMEM (VC) than in those cultured in αMEM (+) or αMEM (-), but this was reversed on day 7. The expression ratios in cells cultured in αMEM (VC)-NT were close to the calcification-induced values in αMEM (+) and αMEM (-) at both time points, and DMEM (VC) consistently yielded relatively lower expression than the other media.

Although the expression of the mid- to late-stage genetic markers *Alpl* and *Bglap* on day 3 differed depending on the medium, there were no medium-dependent differences in expression by day 7. On day 3, *Alpl* expression was significantly higher in the Cal group than in the NT group in αMEM-based medium. The expression of *Bglap* was increased in cells in response to calcification induction in media without fresh VC, and did not significantly change in cells cultured in media containing fresh VC. By day 7, the expression of both genes from cells cultured in medium without fresh VC was significantly increased upon calcification induction compared to cells cultured in medium with fresh VC. Meanwhile, their expression was not changed in cells cultured in medium containing fresh VC. Compared to the NT groups of each culture medium, cells cultured in αMEM (VC) had significantly higher expression of both genes on day 3; however, by day 7, *Alpl* expression was almost the same as on day 3, while the expression of *Bglap* was still predominantly higher in the αMEM (VC) group than that in the other culture media. *Bglap* expression in cells cultured in DMEM (VC) was also higher than that in cells cultured in αMEM (+) and αMEM (-).

The expression of *Col1a2*, which is known to be increased by VC, was significantly increased by day 3 after calcification induction in αMEM (-) and showed an increasing trend in cells cultured in αMEM (+). *Col1a2* expression in cells cultured in αMEM (-)-Cal were comparable to that in αMEM (VC)-NT, to which fresh VC was originally added. By day 7, cells cultured in αMEM (-)-NT showed a 1.5-fold increase in *Col1a2* expression, but the other groups were not affected by calcification induction, and there was no significant difference in the expression for other media.

### 2.5. Simulated Biomaterial Evaluation

**Figure 5:**
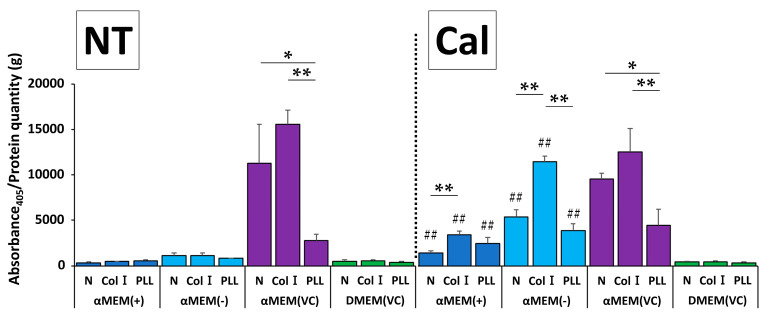
Comparison of alkaline phosphatase (ALP) activity at 7 d after calcification induction using MC3T3-E1 cells in normal (N), Collagen I-coated (Col I), and poly-L-lysine-coated (PLL) culture plates. ALP activity is expressed as Abs_405_/g protein (*n* = 3, * *p* < 0.05, ** *p* < 0.01, ## *p* < 0.01): * comparison among plates under the same media conditions; # comparison between two groups of the same type of nontreated (NT) and calcification (Cal) media in each plate.

We next investigated the effect of different culture media on biomaterial evaluation using two types of biocompatible molecule-coated plates and untreated plates. Only MC3T3-E1 cells cultured in αMEM (VC) without calcification induction showed a significant difference in ALP activity between plates (Figure 5). ALP activity of Col I-coated plate was the highest, but this was not significantly different from that of a normal, non-coated plate. On the other hand, the difference in ALP activity among the plates in response to calcification induction was highest for Col I in all media except DMEM (VC), but no other similarities were observed. The protein concentration of MC3T3-E1 cells cultured in different media in this experiment did not differ among the three types of plates, suggesting no significant difference without αMEM (+)-Cal in cell proliferation (Figure S2).

**Figure 6:**
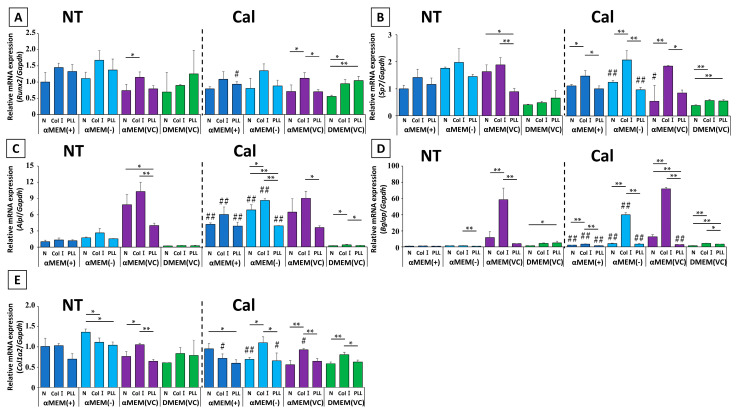
Comparison of the mRNA expression of (**A**) *Runx2*, (**B**) *Sp7*, (**C**) *Alpl*, (**D**) *Bglap*, and (**E**) *Col1a2* at 7 d after calcification induction in MC3T3-E1 cells cultured with different media and different cell culture substrates. Target gene expression was normalized to *Gapdh* expression. The expression levels in MC3T3-E1 cells were compared with MC3T3-E1 cells cultured on nontreated (NT) αMEM (+) on normal (N), non-coated plates (*n* = 3, * *p* < 0.05; ** *p* < 0.01; # *p* < 0.05; ## *p* < 0.01). * Comparison among plates under the same media conditions; ## comparison between two groups of the same NT and calcification (Cal) media in each plate.

Evaluation of gene expression showed several distinct patterns (Figure 6). First, the genes that showed the highest mRNA expression in cells cultured on Col I-coated plates in αMEM-based medium before calcification induction were *Runx2*, *Sp7*, *Alpl*, and *Bglap*. However, in DMEM (VC), coating with poly-L-lysine-coated (PLL) resulted in the highest expression value among all tested genes except *Alpl*. *Col1a2* mRNA showed a different expression pattern ratio in each medium. After calcification induction, the expression patterns of each gene in αMEM-based media tended to be approximately equivalent to untreated condition regardless of whether cells were cultured on coated or non-coated plates. However, there were significant differences in gene expression in cells cultured in DMEM (VC). In these cells, the genes with the highest Col I mRNA expression were *Sp7*, *Alpl*, *Bglap*, and *Col1a2*; only *Runx2* had the same mRNA expression ratio pattern as that on an untreated condition.

## 3. Discussion

Experimental animals are often used to evaluate the efficacy of implant materials for bone tissue regeneration. This method is outmoded compared to in vitro screening of biological safety, as specified in ISO 10993-5, and it is expected that an alternative in vitro method for biological safety screening will soon be developed. In this study, we identified important factors that hinder the development of a new in vitro method.

MC3T3-E1 cells are widely used for evaluating the effects of biomaterials; in Japan, these cells are designated as one of the cell lines used in the “Testing method for biocompatibility of implantable metals using cultured cells” in T0301, which was published in 2000 by Japanese Industrial Standards (JIS) [21]. Therefore, we used MC3T3-E1 cells in this study. αMEM causes MC3T3-E1 cells to proliferate regardless of the cell density, even in αMEM (-) that does not contain VC. Meanwhile, cells cannot proliferate in DMEM that does not contain VC. Although DMEM (VC) containing fresh VC improved cell proliferation at a low cell density, proliferation did not reach the same level as in αMEM (-). Conversely, there was no difference in proliferation between αMEM (+) and αMEM (-), while αMEM (VC) showed a significant increase in cell proliferation compared to αMEM (+) and αMEM (-). These results indicate that VC is not an essential nutrient for MC3T3-E1 cells, although our findings confirm previous reports that VC in commercial liquid medium loses its bioactivity [22,23,24], while fresh VC can enhance cell proliferation [25]. The enhancement of cell proliferation by VC in MC3T3-E1 cells is reportedly due to increased production of collagen and its associated proteins [25,26], but this does not explain the inability of cells to proliferate in DMEM (VC) at low cell densities. DMEM is reportedly less nutrient-rich than αMEM [8,27]; indeed, DMEM expectedly inhibits cell growth at a high cell density. When lysing cells in this study, we confirmed that DMEM (VC) and αMEM (VC) formed equivalent extracellular matrices (data not shown). In addition, the expression of *Col1a2* and *Bglap* was lower in cells cultured in DMEM (VC) than in αMEM (VC) but was higher in DMEM (VC) than in αMEM (+) or αMEM (-). These results suggest that the reported role of VC in MC3T3-E1 cells cannot explain the difference in cell proliferation between αMEM (VC) and DMEM (VC). Thus, in vitro results may differ depending on the base medium, even if the cells and VC conditions are the same.

ALP activity was highest in cells cultured in αMEM (VC) and lowest in cells cultured in DMEM (VC), while *Alpl* gene expression was similar in both media. For other genes, the expression ratios in αMEM (+), αMEM (-), αMEM (VC), and DMEM (VC) were quite different, except for *Col1a2*. These results indicate that the basic properties of MC3T3-E1 cells differ depending on the base culture medium, and the effect of adding VC to the medium also differs.

The results observed before and after induction of calcification with VC and β-glycerophosphate (β-GP), which was used to determine the osteogenic potential of osteoblasts, demonstrate these differences more clearly. Significant changes in the expression of all genes except *Col1a2* occurred at days 3 and 7 after calcification induction in cells grown in αMEM (+) and αMEM (-). These media do not contain fresh VC in the base medium. Significant changes in ALP activity and ARS staining were also observed in cells cultured in αMEM (+) and αMEM (-). Conversely, calcification induction did not cause any significant gene expression changes in cells grown in αMEM (VC), with the exception of *Alpl*. The pattern shown in DMEM (VC) was similar to αMEM (VC). These results indicate that MC3T3-E1 cells cultured in media with fresh VC do not show any additional response to VC-induced calcification induction. ALP activity and gene expression levels in cells grown in αMEM (+) and αMEM (-) after calcification induction are almost the same as the αMEM (VC) values before induction. This result suggests that if αMEM is used immediately after preparation for the culturing MC3T3-E1 cells, the cells remain in a constant state of calcification induction. It has been reported that MC3T3-E1 cells differentiate into bone-like cells without the induction of calcification [26]. The induction of calcification by VC seems to be a compensatory effect for the inactivation of VC by the spread of liquid medium. The MC3T3-E1 cells cultured in αMEM are capable of infinite growth in the absence of VC; however, this observation may not reflect the behavior of osteoblasts in vivo, which require VC. MC3T3 cell proliferation is low in DMEM without VC and high with the addition of VC; thus, these cells may provide a more representative example of the in vivo response. Czekanska et al. reported that normal human primary osteoblasts undergo calcification after calcification induction, even though there is little change in gene marker expression [5]. As shown in Figure S2, MC3T3-E1 cells cultured in DMEM-based media did not show calcification by ARS staining after three weeks; however, calcification occurred by five weeks, and there was little change in gene expression. These findings suggest that calcification occurred in a different pattern than in αMEM-based medium. ALP activity is reportedly lower in cells cultured in DMEM, and calcification is delayed in osteoblasts derived from bone marrow cells compared to that in cells cultured in αMEM or DMEM with VC [8]. This mechanism needs to be further elucidated, along with more detailed biochemical and molecular biological studies of osteoblasts in bone formation in the human body.

Col I- and PLL-coated plates, which have high biocompatibility, were used to clarify the effect of different culture conditions on biomaterial evaluation using MC3T3-E1 cells. Early ALP activity of the cells reportedly increases over time and then plateaus in many cases [5,28,29]. Measurements on day 7 in this study show the accelerating effect of the coating molecules on ALP activity. In this study, Col I, which is known to promote osteogenesis in MC3T3-E1 cells, required an αMEM-based medium supplemented with fresh VC to increase ALP activity on normal plates. However, there was a significant increase in ALP activity compared to normal plates when cells were stimulated with fresh VC after adhering for 24 h in αMEM without fresh VC. MC3T3-E1 cells in αMEM (VC), where VC was added from the start of culture, showed no significant difference in ALP activity between Col I-coated and normal plates. This finding can be interpreted as a difference in the timing of contact of fresh VC with Col I coated on the plate, indicating that the interaction between the material interface and medium that occurs before cellular attachment may influence ALP activity [30]. Simultaneously, we also compared ALP activity of two types of coated plates and their base substrates with the products of a different plate manufacturer to compare the results. ALP activity of the base plate and that of the plate we normally use were different by an order of magnitude. Nearly identical results were obtained even when the plates were remeasured under the same conditions. As such, the culture plates themselves should not be used for comparing biomaterials, since the activity values are completely different depending on the treatment method used by the manufacturer to make the polystyrene plates for adherent cells.

Since it is necessary to consider the fact that some genes undergo temporary changes in expression, it is not desirable to evaluate materials by comparing the expression levels at only one time point. However, differences in gene expression in cells cultured with αMEM and DMEM indicate that media conditions can change the evaluation of the material interface, and that it is essential to establish standard culture conditions, including media that recapitulate in vivo conditions. Differences in gene expression between cells cultured in αMEM (+) and αMEM (-) suggest that the VC originally added to αMEM (+) is oxidized in the medium and has a different effect on the cells than fresh VC. Several studies have already reported the effect of oxidized VC on osteoblasts [22,31]. Since VC is reduced and regenerated by the glutathione-ascorbate cycle in vivo [32,33], it is desirable to use VC 2-phosphate, a VC analog that is stable in aqueous solutions, to evaluate the physiological effects of VC in osteoblast experiments [19,34].

## 4. Materials and Methods

### 4.1. Cells

MC3T3-E1 cells were purchased from Riken BRC (Ibaraki, Japan). The cells were divided into two 6 cm dishes in αMEM (Nacalai Tesque, Kyoto, Japan) supplemented with 10% fetal bovine serum (FBS, Biowest, Nuaille, France) and cultured in a 37 °C incubator with 5% CO_2_. After 24 h, the cells reached 80–90% confluence and were passaged at a density of 4.0 × 10^5^ cells per 10 cm dish every 3 d and stored in Bambanker (Nippon Genetics, Tokyo, Japan). The culture medium used for evaluation was αMEM with VC (αMEM (+), #12571-063; Thermo Fisher Scientific, Waltham, MA, USA), without VC (αMEM (-), #A10490-01, Thermo Fisher Scientific), and Dulbecco’s modified Eagle medium (DMEM, 041-29775; FUJIFILM Wako, Osaka, Japan). FBS was added to each medium to a final percentage of 10%. In addition, αMEM (-) and DMEM which were supplemented with 50 μg/mL VC (Nacalai Tesque) were designated αMEM (VC) and DMEM (VC), respectively. Proliferative tests were performed on cells immediately after thawing. For other experiments, cells were passaged once at a density of 4.0 × 10^5^ cells per 10 cm dish in the respective media. In all experiments—except for the proliferation test—the medium was changed to calcification induction medium supplemented with 100 μg/mL VC and 5 mM β-glycerophosphate (β-GP; Calbiochem, La Jolla, CA, USA) one day after seeding [27,35], and the medium was changed every 3 d. We obtained 48-well plates from Sarstedt (Numbrecht, Germany) and the rest were procured from TrueLine (Nippon Genetics). In the biomaterial simulation experiment, three types of culture plate (IWAKI, Shizuoka, Japan) were used: Collagen type I-coated (Col I; 4820-010), poly-L-lysine-coated (PLL; 4820-020), and normal (N; 3820-024).

### 4.2. Cell Proliferation Test

Thawed cells were seeded in a 96-well plate at densities of 7.5 × 10^3^ cells/mL, 15 × 10^3^ cells/mL, 30 × 10^3^ cells/mL, and 60 × 10^3^ cells/mL. Cell viability was assessed using AlamarBlue^®®^ (Bio-Rad, Hercules, CA, USA). At 24 h, 48 h, and 72 h after the start of the experiment, the media was aspirated, and the cells were incubated with 10% AlamarBlue prepared in Dulbecco’s phosphate-buffered saline (DPBS) at 37 °C for 30 min. Fluorescence intensity was measured on a PlateReader AF2200 (Eppendorf, Hamburg, Germany) with the gain set to 50 (excitation/emission = 535 nm/590 nm).

### 4.3. ALP Activity

Cells were seeded in a 24-well plate at a density of 6.0 × 10^4^ cells/mL. Seven days after induction, media was aspirated, and cells were washed with saline. Cells were lysed by adding 1% NP-40 (Nacalai Tesque), centrifuged at 12,000× *g* for 3 min at 4 °C, and supernatants were collected. Absorbance at 405 nm was measured on a PlateReader AF2200 after 30 min using the ALP assay kit (Takara Bio, Shiga, Japan), according to the manufacturer’s protocol. Protein concentration was measured in the same samples using a BCA assay kit (Nacalai Tesque). ALP activity was expressed as Abs_405_/protein weight (g). For comparisons among plates, the absorbance was measured after 15 min using LabAssay ALP (FUJIFILM Wako).

### 4.4. ARS Staining

Cells were seeded in a 48-well plate at a density of 6.0 × 10⁴ cells/mL. Twenty-one days after the start of calcification, cells were washed then fixed in ice-cold methanol for 20 min. After removing methanol, cells were washed and stained with 1% ARS (pH 6.4) for 5 min to evaluate bone calcification. After staining, plates were washed and dried. For quantification, calcified nodules were dissolved by stirring with 5% formic acid for 10 min. The absorbance of the eluted dye was measured at 405 nm [36].

### 4.5. Real-Time Polymerase Chain Reaction (RT-PCR)

Cells were seeded under the same conditions as for ALP activity. At 3 d and 7 d after induction of calcification, total RNA was extracted with an RNA extraction kit (Nippon Genetics) according to the manufacturer’s protocol. Then, cDNA synthesis was performed using ReverTra Ace qPCR RT Master Mix with gDNA Remover (Toyobo, Osaka, Japan), and real-time PCR was performed using THUNDERBIRD Next SYBR qPCR Mix (Toyobo). Gene expression was measured using the Step One Plus Real-Time PCR System (Thermo Fisher Scientific) and quantified using the ΔΔCt method [37]. The primers used were purchased from Takara Bio and are shown in Table 1.

### 4.6. Statistical Analysis

Data are expressed as the mean ± standard deviation (SD). The Tukey–Kramer method was used to evaluate cell proliferation, and the two-tailed Student’s *t*-test and Tukey–Kramer method were used for comparing the real-time PCR, ALP activity, and ARS staining data. The significance level was set at *p* < 0.05.

## 5. Conclusions

This study revealed that MC3T3-E1 cells cultured in αMEM and DMEM, the two main types of media used in osteoblast experiments, differed greatly in their proliferative, osteogenic, and calcifying capabilities. These differences also affect the evaluation of biomaterials, indicating the possibility of different results even when the same material is evaluated. In addition, VC, which is essential for osteoblast culture, must be added each time to exert its physiological effects. Furthermore, VC that has lost its bioactivity may have unexpected effects on osteoblasts in vitro. Unfortunately, many studies of osteoblasts do not provide clear information on VC, which hinders the understanding of the molecular biology and biochemistry of osteoblasts in bone formation and the reproducibility of experiments. Conversely, we found that it is not necessary to induce calcification with VC. Induction of calcification is thought to be excessive, caused by a lack of basic knowledge about the behavior of VC during the transition of αMEM used in osteoblast research from powder to liquid. In this study, only limited knowledge regarding the direct effects of medium composition on cell behavior and biomaterial activities was gathered; as such, further research is needed to establish in vitro experimental conditions that reflect in vivo bone formation and regeneration and evaluate biomaterials to develop more efficient and accurate hard-tissue implantation strategies. Nonetheless, this work will assist in the fine-tuning of certain experimental designs in terms of the type of base medium, the presence or absence of VC, and medium freshness.

## Figures and Tables

**Table 1:** Osteoblast markers assessed by real time PCR.

Takara Bio Primer_Set ID	Symbol	Name
MA050371	*Gapdh*	Glyceraldehyde-3-phosphate dehydrogenase
MA144435	*Runx2*	Runt-related transcription factor 2
MA147894	*Sp7*	Sp7 transcription factor (Osterix)
MA127137	*Alpl*	Alkaline phosphatase, biomineralization associated
CH000874	*Bglap*	Bone gamma-carboxyglutamate protein (Osteocalcin)
MA128559	*Col1a2*	Collagen type I alpha 2 chain

## Data Availability

All data are available from the corresponding author upon reasonable request.

## Supplementary Materials

The following are available online at https://www.mdpi.com/article/10.3390/ijms22147752/s1, Figure S1: Effect of calcification of MC3T3-E1 cells after 21 and 35 days. Figure S2: Comparison of protein concentration at 7 days using MC3T3-E1 cells using three types of plate.

## References

[1] Im G.I. (2020). Biomaterials in orthopaedics: The past and future with immune modulation. Biomater. Res..

[2] Wang G., Zreiqat H. (2010). Functional Coatings or Films for Hard-Tissue Applications. Materials.

[3] Qin L., Yao S., Zhao J., Zhou C., Oates T.W., Weir M.D., Wu J., Xu H.H.K. (2021). Review on Development and Dental Applications of Polyetheretherketone-Based Biomaterials and Restorations. Materials.

[4] Kohli N., Ho S., Brown S.J., Sawadkar P., Sharma V., Snow M., García-Gareta E. (2018). Bone remodelling in vitro: Where are we headed?: -A review on the current understanding of physiological bone remodelling and inflammation and the strategies for testing biomaterials in vitro. Bone.

[5] Czekanska E.M., Stoddart M.J., Ralphs J.R., Richards R.G., Hayes J.S. (2014). A phenotypic comparison of osteoblast cell lines versus human primary osteoblasts for biomaterials testing. J. Biomed. Mater. Res. A.

[6] Czekanska E.M., Stoddart M.J., Richards R.G., Hayes J.S. (2012). In search of an osteoblast cell model for in vitro research. Eur. Cell Mater..

[7] Hinoi E., Fujimori S., Takemori A., Yoneda Y. (2002). Cell death by pyruvate deficiency in proliferative cultured calvarial osteoblasts. Biochem. Biophys. Res. Commun..

[8] Coelho M.J., Cabral A.T., Fernande M.H. (2000). Human bone cell cultures in biocompatibility testing. Part I: Osteoblastic differentiation of serially passaged human bone marrow cells cultured in alpha-MEM and in DMEM. Biomaterials.

[9] Brzezińska O., Łukasik Z., Makowska J., Walczak K. (2020). Role of Vitamin C in Osteoporosis Development and Treatment-A Literature Review. Nutrients.

[10] Chin K.Y., Ima-Nirwana S. (2018). Vitamin C and Bone Health: Evidence from Cell, Animal and Human Studies. Curr. Drug Targets.

[11] Jonason J.H., O’Keefe R.J. (2014). Isolation and culture of neonatal mouse calvarial osteoblasts. Methods Mol. Biol..

[12] Bakker A.D., Klein-Nulend J. (2012). Osteoblast isolation from murine calvaria and long bones. Methods Mol. Biol..

[13] Orriss I.R., Taylor S.E., Arnett T.R. (2012). Rat osteoblast cultures. Methods Mol. Biol..

[14] Doolittle M.L., Ackert-Bicknell C.L., Jonason J.H. (2021). Isolation and Culture of Neonatal Mouse Calvarial Osteoblasts. Methods Mol. Biol..

[15] Kodama H., Amagai Y., Sudo H., Kasai S., Yamamoto S. (1981). Establishment of a clonal osteogenic cell line from newborn mouse calvaria. Jpn. J. Oral Biol..

[16] Hiura K., Sumitani K., Kawata T., Higashino K., Okawa M., Sato T., Hakeda Y., Kumegawa M. (1991). Mouse osteoblastic cells (MC3T3-E1) at different stages of differentiation have opposite effects on osteoclastic cell formation. Endocrinology.

[17] Zhou H.Y., Takita H., Fujisawa R., Mizuno M., Kuboki Y. (1995). Stimulation by bone sialoprotein of calcification in osteoblast-like MC3T3-E1 cells. Calcif. Tissue Int..

[18] Wang D., Christensen K., Chawla K., Xiao G., Krebsbach P.H., Franceschi R.T. (1999). Isolation and characterization of MC3T3-E1 preosteoblast subclones with distinct in vitro and in vivo differentiation/mineralization potential. J. Bone Miner. Res..

[19] Dillon J.P., Waring-Green V.J., Taylor A.M., Wilson P.J., Birch M., Gartland A., Gallagher J.A. (2012). Primary human osteoblast cultures. Methods Mol. Biol..

[20] Bilousova G., Jun d.H., King K.B., De Langhe S., Chick W.S., Torchia E.C., Chow K.S., Klemm D.J., Roop D.R., Majka S.M. (2011). Osteoblasts derived from induced pluripotent stem cells form calcified structures in scaffolds both in vitro and in vivo. Stem. Cells.

[21] Testing Method for Biocompatibility of Implantable Metals Using Cultured Cells 2000. https://www.jisc.go.jp/app/jis/general/GnrJISSearch.html.

[22] Takamizawa S., Maehata Y., Imai K., Senoo H., Sato S., Hata R. (2004). Effects of ascorbic acid and ascorbic acid 2-phosphate, a long-acting vitamin C derivative, on the proliferation and differentiation of human osteoblast-like cells. Cell Biol. Int..

[23] Roach H.I., Hillier K., Shearer J.R. (1985). Stability of ascorbic acid and uptake of the vitamin by embryonic chick femurs during long-term culture. Biochim. Biophys. Acta.

[24] Feng J., Melcher A.H., Brunette D.M., Moe H.K. (1977). Determination of L-ascorbic acid levels in culture medium: Concentrations in commercial media and maintenance of levels under conditions of organ culture. In Vitro.

[25] Harada S., Matsumoto T., Ogata E. (1991). Role of ascorbic acid in the regulation of proliferation in osteoblast-like MC3T3-E1 cells. J. Bone Miner. Res..

[26] Sudo H., Kodama H.A., Amagai Y., Yamamoto S., Kasai S. (1983). In vitro differentiation and calcification in a new clonal osteogenic cell line derived from newborn mouse calvaria. J. Cell Biol..

[27] Orriss I.R., Hajjawi M.O., Huesa C., MacRae V.E., Arnett T.R. (2014). Optimisation of the differing conditions required for bone formation in vitro by primary osteoblasts from mice and rats. Int. J. Mol. Med..

[28] Beck G.R., Sullivan E.C., Moran E., Zerler B. (1998). Relationship between alkaline phosphatase levels, osteopontin expression, and mineralization in differentiating MC3T3-E1 osteoblasts. J. Cell Biochem..

[29] Franceschi R.T., Iyer B.S. (1992). Relationship between collagen synthesis and expression of the osteoblast phenotype in MC3T3-E1 cells. J. Bone Miner. Res..

[30] Khan M.R., Mordan N., Parkar M., Salih V., Donos N., Brett P.M. (2019). Atypical Mesenchymal Stromal Cell Responses to Topographic Modifications of Titanium Biomaterials Indicate Cytoskeletal- and Genetic Plasticity-Based Heterogeneity of Cells. Stem Cells Int..

[31] Qutob S., Dixon S.J., Wilson J.X. (1998). Insulin stimulates vitamin C recycling and ascorbate accumulation in osteoblastic cells. Endocrinology.

[32] Wells W.W., Xu D.P. (1994). Dehydroascorbate reduction. J. Bioenerg. Biomembr..

[33] Noctor G., Foyer C.H. (1998). ASCORBATE AND GLUTATHIONE: Keeping Active Oxygen Under Control. Annu. Rev. Plant Physiol. Plant Mol. Biol..

[34] Hata R., Senoo H. (1989). L-ascorbic acid 2-phosphate stimulates collagen accumulation, cell proliferation, and formation of a three-dimensional tissuelike substance by skin fibroblasts. J. Cell Physiol..

[35] Huang B., Wang Y., Wang W., Chen J., Lai P., Liu Z., Yan B., Xu S., Zhang Z., Zeng C. (2015). mTORC1 Prevents Preosteoblast Differentiation through the Notch Signaling Pathway. PLoS Genet..

[36] Miyazaki T., Miyauchi S., Tawada A., Anada T., Matsuzaka S., Suzuki O. (2008). Oversulfated chondroitin sulfate-E binds to BMP-4 and enhances osteoblast differentiation. J. Cell Physiol..

[37] Livak K.J., Schmittgen T.D. (2001). Analysis of relative gene expression data using real-time quantitative PCR and the 2(-Delta Delta C(T)) Method. Methods.

