# Supplementary material for: Evaluation of MC3T3-E1 Cell Osteogenesis in Different Cell Culture Media
Source: Int J Mol Sci. 2021 Jul 20;22(14):7752. doi: 10.3390/ijms22147752 (PMC8304275; doi:10.3390/ijms22147752)
Supplement: Supplementary file 1 [file ijms-22-07752-s001.zip › ijms-1281633-supplementary.pdf]

Supplementary Information

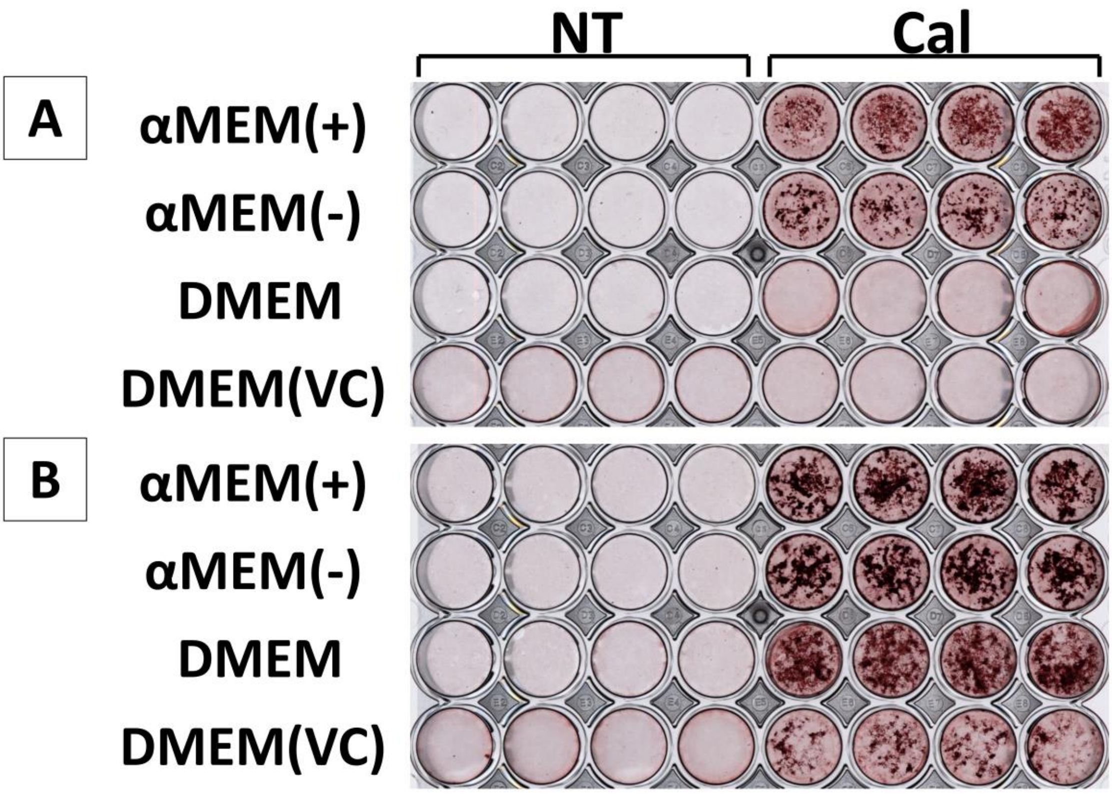

Figure S1 Effect of calcification on MC3T3-E1 cells after 21 d (A) and 35 d (B). After thawing, MC3T3-E1 cells were passaged twice at 4.0 × 10^5 cells per 10 cm dish every 3 d in αMEM (+), and cells were seeded at a density of 6.0 × 10^4 cells/mL in a 48-well plate, with four wells of non-treated (NT) and four wells of calcification induction (Cal) per condition. Alizarin Red S (ARS) staining was performed to evaluate calcification after 21 d and 35 d.

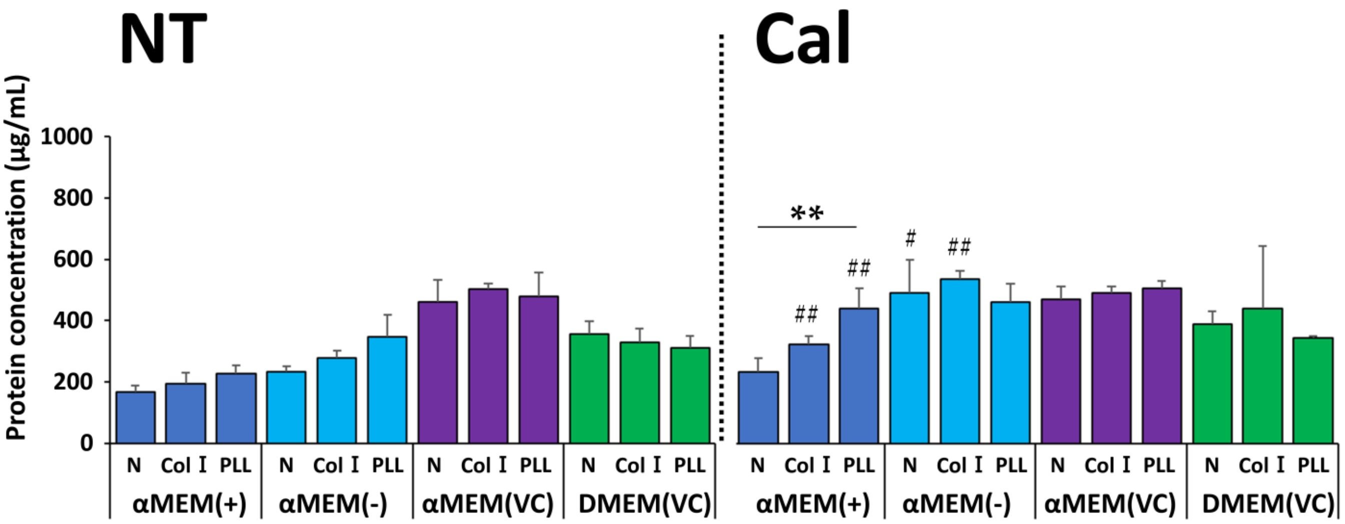

Figure S2 Comparison of protein concentration at 7 days after calcification induction using MC3T3-E1 cells in normal (N), collagen I-coated (Col I), and poly-L-Lysine-coated (PLL) culture plates (n = 3, ** $p < 0.01$, $^{\#} p < 0.05$, $^{\#\#}$

$p < 0.01$): *Comparison among plates under the same media conditions, #Comparison between two groups of the same type of nontreated (NT) and calcification (Cal) media in each plate.
